# Perioperative fatal embolic cerebrovascular accident after radical prostatectomy

**DOI:** 10.4103/2152-7806.65055

**Published:** 2010-07-01

**Authors:** Ramsis F. Ghaly, Kenneth D. Candido, Nebojsa Nick Knezevic

**Affiliations:** 1 Department of Anesthesiology, Advocate Illinois Masonic Medical Center, Chicago, IL 60657, USA; 2 Ghaly Neurosurgical Associates, Aurora, IL 60504, USA

**Keywords:** Cerebrovascular accident, fatal, perioperative, radical prostatectomy

## Abstract

**Background:**

There is little written about the management of perioperative cerebrovascular accident (CVA). To the best of our knowledge, the present case report represents the first case in the literature of a well-documented intraoperative embolic CVA and perioperative mortality in a relatively healthy, young patient with no contributing comorbidity and no noteworthy intraoperative event.

**Case Description:**

A 53-year-old man presented for radical prostatectomy under general anesthesia. The anesthetic course and procedure were uneventful. In the postanesthesia care unit (PACU), the patient was moving all extremities but was still sedated. One hour later, he developed left hemiplegia, facial dropping, slurred speech and his head was turned to the right. The next day his mental status deteriorated, and on an emergency basis he was intubated. A CT scan of the head showed a malignant hemispheric right cerebrovascular accident with leftward midline shift. Even aggressive treatment, including a right decompressive hemicraniectomy, could not lower the high intracranial pressure, and the patient expired on the third postoperative day.

**Conclusion:**

Guidelines for identifying and treating perioperative hemispheric CVA are urgently needed, with modification of the antiquated and useless criterion of “patient seen neurologically normal at induction time” to more useful objective criteria including “intraoperative neurophysiological recording change, gross extremity movements, facial dropping, follows simple commands” while excluding a drug-induced, sedative-influenced globally-impaired cognitive state that may last for hours.

## INTRODUCTION

Cerebrovascular accidents (CVAs) are the second leading cause of death worldwide. It is estimated that more than 600,000 Americans are newly diagnosed yearly with stroke; one every 53 seconds, with one dying every 3.3 minutes.[3] Perioperative CVA is a rare phenomenon, and the risk in noncardiac surgery and in patients 50 to 70 years old is estimated to be 0.2% to 0.4%.[13,15] Larsen *et al*.[15] showed that CVA usually occurred 5 to 26 days after noncardiac, noncarotid artery surgery, and these cases were not directly correlated to surgery and anesthesia.

Prostate cancer is the most commonly diagnosed cancer in males.[12] Begg *et al*.[4] found in 11,522 males undergoing radical prostatectomy that the major postoperative complications included those related to cardiac pathology (5.5%), vascular pathology (4.7%), wound infection (2.6%) and the genitourinary system (2.8%). However, mortality after open radical prostatectomy is relatively low. Alibhai *et al*.[2] found that the 30-day mortality rate following open radical prostatectomy was 0.5% (53 out of 11,010 patients) for otherwise healthy men up to age 79 years.

There is little written about the management of perioperative CVA. To the best of our knowledge, the present case report represents the first case in the literature of a well-documented intraoperative embolic CVA and perioperative mortality in a relatively healthy, young patient with no contributing comorbidity and no noteworthy intraoperative event.

## CASE REPORT

A 53-year-old man, American Society of Anesthesiologists (ASA) physical status class II, presented for radical prostatectomy for prostatic adenocarcinoma without signs of metastasis. Past medical history included the following: diet-controlled diabetes, benign hypertension, well-controlled hypercholesterolemia, history of hepatitis and cholecystitis. Induction of anesthesia was achieved using fentanyl 2 mcg/kg, propofol 3 mg/kg and succinylcholine 2 mg/kg, followed by tracheal intubation. Anesthesia was maintained using sevoflurane 1 monitored anesthesia care (MAC) with controlled ventilation. Baseline blood pressure was 124/70 mm Hg, and systolic pressure was maintained at 100 mm Hg throughout the course of surgery. Radical retropubic prostatectomy and pelvic lymphadenectomy were performed. Frozen sections revealed no malignancy in the lymph nodes. Estimated blood loss was 500 mL and no transfusion was required. The patient was extubated in satisfactory condition while still in the operating room.

In the postoperative care unit (PACU), the patient could converse normally and was moving all extremities but was still mildly sedated. One hour later, he was found to have a left hemiplegia, facial dropping, slurred speech and his head was turned to the right. Pupils were equal and reactive. He was unable to stick his tongue out but was able to state that he was in a hospital. The patient had normal strength in the right arm and right leg. Neurology consultation recommended “no definitive therapy” because more than 8 hours had elapsed from the induction time, wherein the patient was found to be neurologically intact. Contributory factors for stroke were not present, which included obesity, smoking, family history of stroke, history of stroke or transient ischemic episodes (TIEs), intracranial cerebrovascular disease, noncontrolled hypertension, extracranial carotid stenosis, ulcerative atherosclerotic plaques, noncontrolled hyperlipidemia, peripheral vascular diseases, noncontrolled diabetes mellitus, sickle cell disease, cardiac causes (e.g., atrial fibrillation, myocardial infarction, atrial myoma, ventricular aneurysm, prosthetic heart valve, heart failure, coronary artery disease), hypercoagulable state (protein S and C deficiency, Factor V deficiency, antithrombin III, lupus anticoagulant, antiphospholipid antibody, anticardiolipin, homocysteinurea).[7] Perioperative factors related to anesthesia included type of surgery (e.g., cerebrovascular and cardiovascular), perioperative hemodynamic instability, prolonged hypotension or hypertension, central venous internal jugular vein catheterization, conditions of elevated airway pressure in face of potential patent foramen ovale.[7]

**Figure 1:**
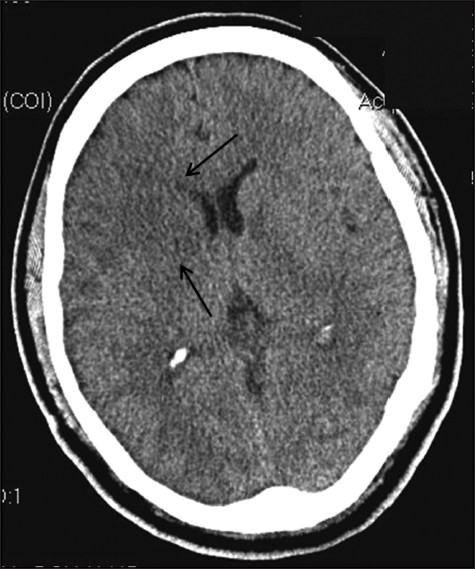
Axial CT scan of the brain without contrast 2 hours after completion of prostatectomy demonstrates early lucency of evolving infarction in the right MCA territory

**Figure 2:**
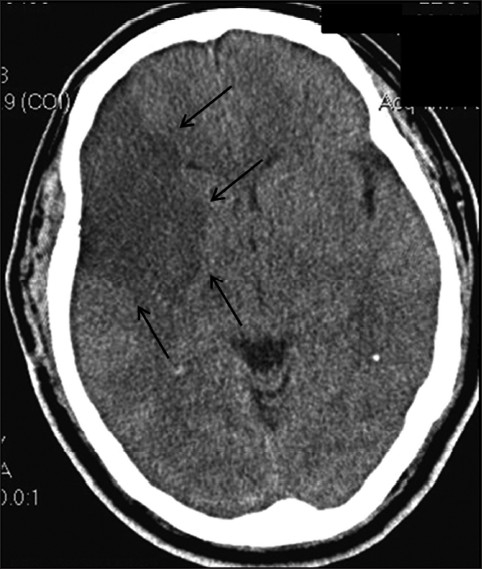
Axial CT scan of the brain without contrast 20 hours after completing prostatectomy demonstrates right MCA territory infarction with 3-mm midline shift

**Figure 3:**
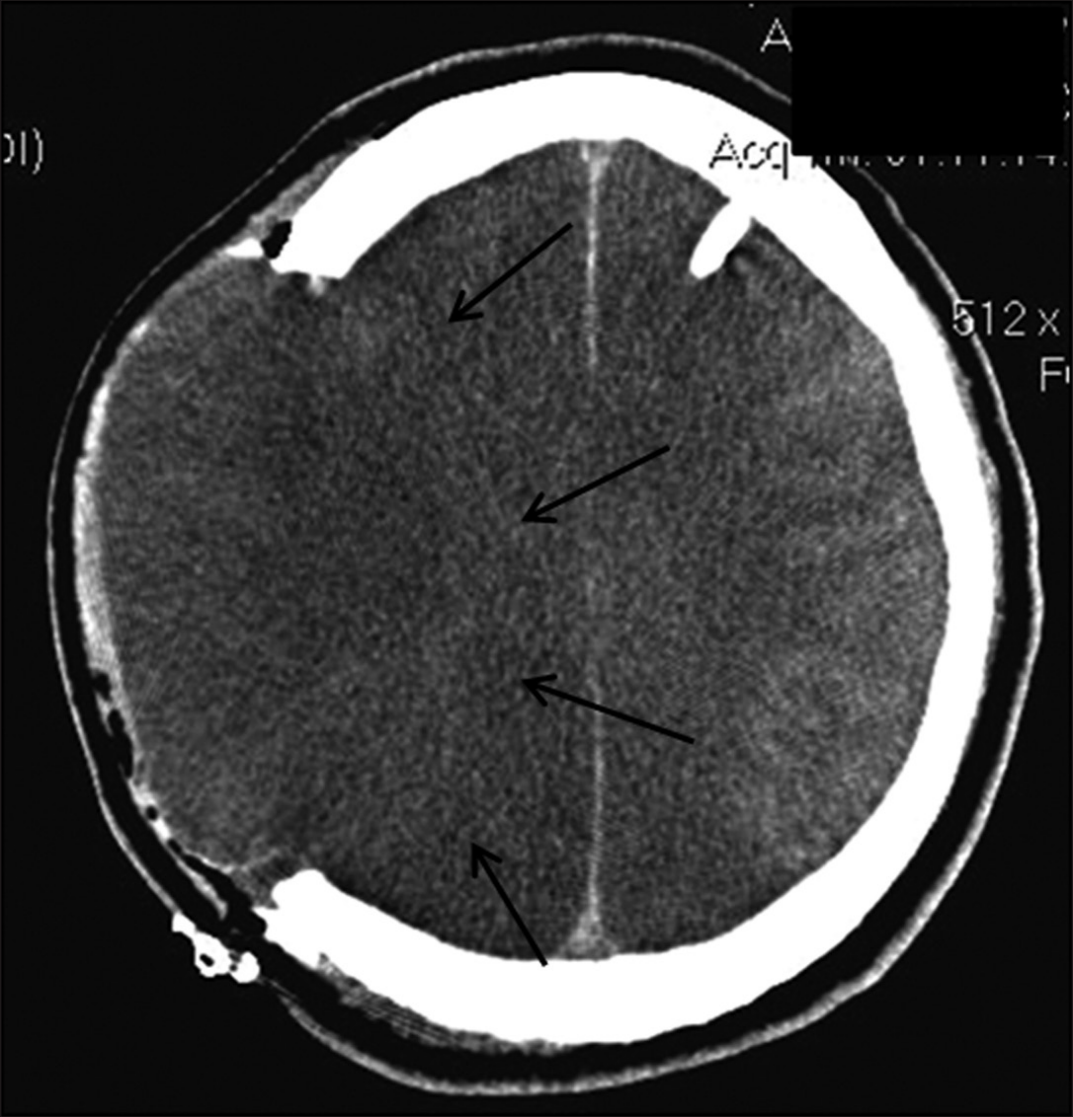
Axial CT scan showing total right hemispheric infarction, herniation of the brain through the defect, massive bihemispheric swelling, loss of white-gray matter differentiation

Serial CT scans of the head were performed, which showed a right-sided cerebral infarction without mass effect [Figure 1]. The following day his mental status deteriorated, and he was urgently intubated. Mannitol was administered, and CT scans of the head showed malignant hemispheric right CVA with a 3-mm [Figure 2] and later an 8-mm leftward midline shift and small left frontal swelling. Right decompressive hemicraniectomy was performed, and an intracranial pressure (ICP) monitor was placed. Initial ICP was 11 mm Hg. Repeat CT scans after surgery showed extensive edema and swelling of both hemispheres [Figure 3]. On postoperative day 3, nuclear imaging demonstrated absence of cerebral blood flow, and the patient was declared brain dead by nuclear medicine criteria. The ICP rose to 70 mm Hg on postoperative day 3, and the patient expired later that day.

**Figure 4:**
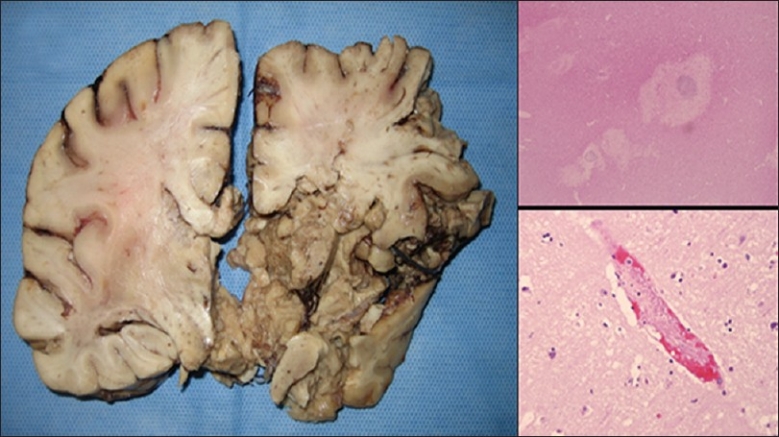
a) Autopsy brain section demonstrates extensive right temporal lobe softening caused by acute infarction; b) pathohistological slide (hematoxylin and eosin staining) showing necrosis, inflammation, neuronal loss due to severe ischemia; c) pathohistological slide (hematoxylin and eosin staining) showing blood vessel with fibrin thrombus

At autopsy, the brain demonstrated non-hemorrhagic thromboembolic cerebrovascular disease and an acute infarct in the territory of the right middle cerebral artery (MCA) [Figure 4]. The patient had increased intracranial pressure, central as well as left and right uncal herniations with acute cerebral and brainstem hypoxic/ ischemic injuries. Massive diffuse brain swelling with herniation through the skull defect was also noted. Mild atherosclerotic cardiovascular disease and systemic disease were seen without occlusive disease including the carotids. There was diffuse pulmonary congestion and mild pulmonary edema. There were hypertensive changes in the heart (ventricular hypertrophy, mild atrial dilatation) with no acute event noted. There was no evidence of thrombosis in the pelvic and iliac veins.

## DISCUSSION

One of the serious risks of prostatectomy is deep venous thrombosis (DVT), with an incidence rate ranging from 1% to 4%, with pulmonary embolism noted in 0.5% of cases.[11] DVT is considered the most common cause of increased postoperative morbidity and mortality in patients with pelvic and prostatic cancer. Hemostatic factors in patients undergoing radical prostatectomy usually indicate a baseline hypercoagulable state.[14] In addition to an anesthesia-induced hypercoagulable state, venous stasis and surgical manipulation may result in thrombosis or dislodgment of a plaque.[13] However, an autopsy performed on this patient demonstrated that the cause of the CVA was an embolic MCA ischemic stroke. Iliac and pelvic veins showed no thrombus formation. Laboratory results did not reveal a hypercoagulable state. The patient’s risk factors for thromboembolic stroke were minimal (elevated but well-controlled cholesterol; mild hypertension; borderline diabetes mellitus; and asymptomatic diffuse atherosclerosis with minimal intravascular Plaques were not hemodynamically significant). Potential causes of embolism may include acute venous thrombus formation in the lower extremities, deep pelvic veins or in the stretched iliac veins by the surgery, which under positive pressure ventilation might embolize to the MCA and cause acute CVA. Furthermore, the heart demonstrated no persistent patent foramen ovale to facilitate a right-to-left shunt.[9] However, the autopsy showed a very rare congenital anomaly of the left coronary artery, which may be associated with ischemia of the left ventricle that might promote an arrhythmic event resulting in sudden death or which may remain silent and asymptomatic for extended periods of time.[5]

The patient expired on postoperative day 3 despite hemispheric decompressive hemicraniectomy. Massive hemispheric cerebral infarct usually progresses to “malignant brain swelling” after a sudden interruption of the entire MCA territory, with a mortality rate of 80% in the first 3 to 5 days post ictus.[17] Current guidelines include obtaining a CT scan of the head within 25 minutes of the onset of signs or symptoms of CVA. MRI perfusion-diffusion mismatch and CT perfusion-blood volume mismatch are on the horizon to assure quantitatively better therapeutic modalities.[1]

The time for identifying a CVA is essential to begin effective anticoagulant therapy; for intravenous tissue plasminogen activator (tPA), this time limit is 3 hours; for other “stroke-rescue” procedures such as intra-arterial tPA and embolus retrieval mechanically, this time limit is 6 to 8 hours.[1] The goal is to reestablish blood flow that has been acutely interrupted, before irreversible infarction and necrosis occur. If blood flow is not resumed and if the embolus is not removed, an ischemic cascade will activate necrotic and apoptotic pathways, cellular failure, release of neurotoxic agents and the formation of free radicals. Hemispheric infarction will occur, and subsequent malignant brain swelling with a refractory high ICP will result within 3 days post ictus. Brain swelling peaks at 3 to 5 days, and death results from transtentorial herniation.[10] Herniation is more obvious in younger individuals and proceeds more quickly to death.[16] Unfavorable prognostic factors include the following: hypodensity on CT scan (>50% of MCA territory), midline shift >4 mm, a large-volume infarct, and displacement of the septum pellucidum.[6,16,18]

An obvious limitation is to identify the last time that the patient was “neurologically normal.” If a CVA occurred while asleep or under anesthesia, then the clock starts when the patient went to sleep. For a perioperative CVA, this time is represented by the time of induction of anesthesia. Perioperative CVA therapy was withheld in this patient due to the assumption that the patient presented as neurologically “normal” at induction time, and 8 hours had already elapsed at extubation, despite the fact that in the PACU, on admission the patient was moving all extremities. The current recommendation for stroke-rescue procedures to be done beyond the 8-hour window is considered as investigatory only.[1] However, a modification of these assumptions may be necessary because under anesthesia the brain is protected by anesthetics, as well as by relative hypothermia and the suppression of metabolic activity and oxygen consumption. Sedatives and inhaled anesthetics are known to distort cognitive functions for hours or even days.[8] Anesthetics may also cause permanent subtle or overt personality changes, particularly in elderly patients.[8]

General anesthesia is typically recommended for radical prostatectomy; however, regional block may have the advantage of promoting blood flow to the extremities, preventing venous stasis by enhancing blood rheology, which will not affect mental status during neurological examination. Nonetheless, the inability to assess lower extremity motor function following the regional block may be troubling; and if spinal subarachnoid blockade is performed, this may expedite the process of brain herniation in susceptible patients.

Guidelines for identifying and treating perioperative hemispheric CVA are urgently needed, with modification of the antiquated and useless criterion of “patient seen neurologically normal at induction time,” to more useful objective criteria including “intraoperative neurophysiological recording change, gross extremity movements, facial dropping, follows simple commands” while excluding a drug-induced, sedative-influenced globally-impaired cognitive state that may last for hours.
